# Biochemical Characterization and Storage Stability of Process Waters from Industrial Shrimp Production

**DOI:** 10.1021/acsomega.1c03304

**Published:** 2021-11-10

**Authors:** Bita Forghani, Ann-Dorit Moltke Sørensen, Gustaf Fredeus, Kenneth Skaaning, Johan Johannesson, Jens J. Sloth, Ingrid Undeland

**Affiliations:** † Food and Nutrition Science, Biology and Biological Engineering, Chalmers University of Technology, Gothenburg 412 96, Sweden; ‡ National Food Institute, Technical University of Denmark, Kgs. Lyngby 2800, Denmark; § Räkor & Laxgrossisten AB, Gothenburg 42132, Sweden

## Abstract

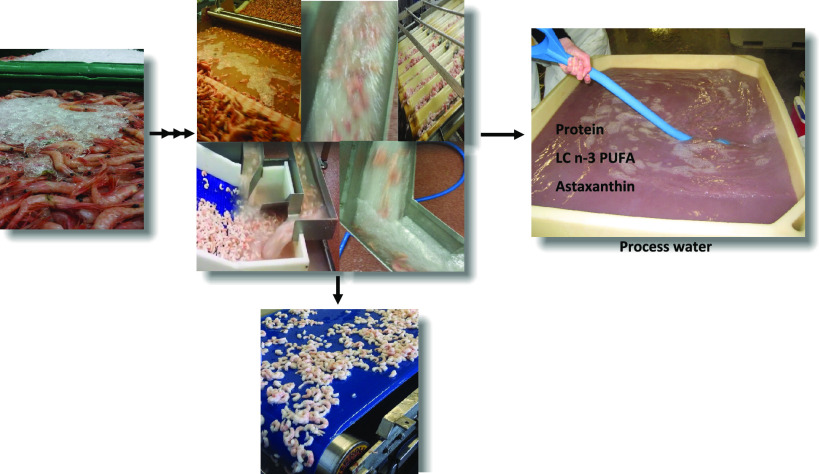

Shrimp boiling water (SBW) and shrimp peeling water (SPW), generated during shrimp processing, were characterized in terms of crude composition, volatile compounds, as well as nutritional and potentially toxic elements over a 13 month sampling period. The storage stability of both waters was also evaluated. Results showed that SBW contained on median 14.8 g/L protein and 2.2 g/L total fatty acids with up to 50% comprising eicosapentaenoic acid (EPA) and docosahexaenoic acid (DHA). Astaxanthin esters, which dominated the total astaxanthin, were 2.8 mg/L on median. SPW, on the other hand, contained on median 1.0 g/L of protein, 0.21 g/L of total fatty acids, and 1.2 mg/L astaxanthin esters. For both side-streams, essential amino acids were up to 50% of total amino acids. For SBW and SPW, the most abundant nutritional elements were Na, K, P, Ca, Cu, and Zn. The contents of all potentially toxic elements were below the detection limits, except for As. SBW was more stable at 4 °C compared to SPW as shown, e.g., by thiobarbituric acid reactive substances and relative changes in total volatile basic nitrogen. The extensive compositional mapping of SBW/SPW provides crucial knowledge necessary in the exploitation and value-adding of such side-streams into food or feed products.

## 1 Introduction

Half of the industrial pollution in the world originates from food industries, and among them, seafood processing companies contribute greatly by generating large amounts of wastewaters.^1^ This is due to water being a crucial tool in seafood processing steps such as thawing, cooling, filleting, peeling, transportation, storage, marination, and cooking that yield significant loads of organic and inorganic compounds in the used process waters. The diverse nature of different types of seafood process waters will lead to different sets of challenges and costs in the path of cleaning and to different degrees of pollution in countries where wastewaters are still allowed to be directly released into the ocean. At the same time, many of the compounds leaching out from the seafood tissue to process waters are of potential high value, such as proteins, peptides, free amino acids, nutritional elements, antioxidants, and long-chain n-3 polyunsaturated fatty acids (LC n-3 PUFA), calling for changed habits.

In line with the UN sustainable development goals (SDGs), food industries strive to move toward zero waste and efficient production using incoming raw materials to the fullest. One way to lessen the raw material loss connected to process waters is to recover the dissolved nutrients while waters are still food grade, thereby allowing them to be maintained in the food chain and converted into food or feed ingredients. To properly design the challenging task of recovering leached nutrients, the generated process waters must first be characterized to gain enough knowledge on the quantity and nature of inherent compounds. Indeed, information about process water volumes is also crucial to identify a specific recovery approach.

In a typical processing line producing boiled and peeled shrimps, up to 65 m^3^ water is used per tonne of final peeled shrimp. Process waters are generated during the steaming and peeling steps, but not least during the transportation of shrimps in between these steps. There are thus strong incentives to minimize nutrient losses taking place along with the current treatments of these massive amounts of water as wastewaters. So far, a few studies have reported on the recovery of volatile compounds, bioactive peptides, and astaxanthin from shrimp process waters by employing techniques such as ultrafiltration, reverse osmosis, and nano filtration together with osmotic evaporation.^2−8^ In addition, our own group has reported on flocculation combined with flotation to recover protein-enriched biomasses from shrimp boiling water (SBW) and peeling water (SPW).^9,10^ However, still very little is known about the variations in the nutrient composition of shrimp process waters over an extended production period. A broad nutrient characterization as well as insight into potential toxic elements and shelf life would aid the implementation of new and already reported recovery tools for proteins, fatty acids, antioxidants, and flavor molecules.

In the present study, our aim was to map the crude composition and profiles of polypeptides, amino acids, fatty acids, volatile compounds, and astaxanthin as well as the nutritional and potential toxic elements of SBW and SPW over a 13 month period with monthly samplings. The storage stability of selected SPW and SBW was also investigated using sensory, microbial, and lipid oxidation analyses.

## 2 Material and Methods

### 2.1 Materials

Five liters each of SBW and SPW generated during shrimp, *Pandalus borealis,* boiling and peeling was sampled monthly from October 2016 to November 2017 on Friday mornings between 8:00 and 9:00 a.m. at Räkor & Laxgrossisten AB, Gothenburg, Sweden. The SBW was sampled at a point immediately after the steaming step, and the SPW was sampled right after the peeling step, before the water leaves the processing line. The latter is thus a pool of all types of process waters including SBW, transport water, and peeling water.

### 2.2 pH, Dry Matter, and Ionic Strength

pH was measured at 20 °C with an M210 standard pH meter (Radiometer Analytical, Lyon, France). Ionic strength (IS) was measured using a conductivity meter (Radiometer Analytical, Lyon, France) and was calculated against a standard curve of NaCl in percentage. Dry matter was determined based on a gravimetric method comprising the pre-weighed samples being dried in a 105 °C oven (Electrolux, Stockholm, Sweden) until a constant weight was obtained. Dry matter was calculated using the following formula:
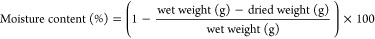


### 2.3 Protein Content and Polypeptide Profiling Using Sodium Dodecyl Sulfate-Polyacrylamide Gel Electrophoresis (SDS-PAGE)

Protein has been measured following the method of Lowry et al.^11^ modified by Markwell et al.^12^ using serum bovine albumin as standard in the concentration range of 10–100 μg/mL. Absorbance was read at 660 nm using a Cary60 BIO UV–vis spectrophotometer (Varian Australia Pty. Ltd., Victoria, Australia). The polypeptide profile of SBW collected over the 13 month period was determined using SDS-PAGE according to the method of Laemmli.^13^ Electrophoresis was carried out using Mini-protean TGX 4–20% pre-cast gels (Bio-Rad Laboratories, USA). Briefly, SBW samples were mixed 1:1 (v/v) with the loading dye and 20 μg protein was loaded into each lane. The polypeptide molecular standard was a broad range (10–250 kDa). Protein bands were stained by Coomassie Brilliant blue G-250. SPWs were not subjected to SDS-PAGE due to the very low protein content.

### 2.4 Total and Free Amino Acid Content

The amino acid composition (free and total) in the process waters was determined by HPLC-MS. For analysis and determination of total amino acids, the process waters were hydrolyzed and derivatized using an EZfaast amino acid kit (Phenomenex, Torrance, CA, USA). The acid hydrolysis was applied to release the amino acids and comprised 6 M HCl and heat treatment (1 h, 110 °C) using a microwave (Multiwave 3000, Anton Paar GmbH, Graz, Austria). The subsequent neutralized samples were purified by a solid-phase extraction sorbent tip, and derivatization was performed before the injection of sample aliquots into an Agilent HPLC 1100 instrument (Santa Clara, CA, USA) coupled to an Agilent ion trap mass spectrometer (MS). For the analysis and determination of free amino acids, the process waters were derivatized. The amino acids were identified by comparing the retention time and mass spectra of an external standard mixture. Calibration curves were prepared and analyzed by HPLC-MS for quantification.

### 2.5 Fatty Acid Content and Composition

Fatty acid analysis using gas chromatography (GC)–MS was performed after extraction of lipids according to Lee et al^14^ and subsequent methylation according to Lepage and Roy^15^ with some modifications. The extraction was performed using chloroform–methanol (1:2), and C17 was added as an internal standard followed by vortexing for 10 s and addition of 0.5% NaCl to reach the ratio of 1:2.75 (v/v, water phase/chloroform–methanol). Following phase separation, chloroform was evaporated at 40 °C. Methylation was conducted by addition of 2 mL of toluene and 2 mL of acetylchloride/methanol (1:10), and the solution was incubated at 60 °C for 120 min. One milliliter of Milli-Q water (conductivity of 18 Ω/cm^–1^) and 2 mL of petroleum ether were added to the tubes, which were vortexed for 10 s thereafter and centrifuged at 2500*g* for 5 min. The upper phase was transferred to a new tube and evaporated under nitrogen at 40 °C. Evaporated samples were then dissolved in 0.5 mL of isooctane. Identification and quantification of fatty acids were carried out by GC–MS using an Agilent Technologies 7890 A GC system connected to an Agilent Technologies 5975 inert MSD (Kista, Sweden) as described elsewhere.^16^ Total fatty acids were calculated as the sum of all measured fatty acids in the sample minus the internal standard.

### 2.6 Volatile Compound Analysis

Collection of volatile compounds was performed by dynamic headspace ″purge and trap″. Volatiles from the process waters (4 g) were purged (37 °C) with nitrogen (260 mL/min) for 30 min and trapped on Tenax tubes. Trapped volatiles were desorbed and separated on GC (Agilent Technologies 6890N, CA, USA) with a DB1701 column (30 m; i.d. 0.25 mm; 1 μm film thickness; Agilent Technologies). The oven program had an initial temperature of 45 °C for 5 min, and the temperature was increased gradually by 1.5 °C/min until 55 °C, then by 2 °C/min until 90 °C, and finally by 8 °C/min until 230 °C, where the temperature was held for 8 min. The individual volatiles were analyzed by MS (Agilent 5973 Network Mass Selective Detector, Agilent Technologies; electron ionization mode, 70 eV; *m/z* scan between 30 and 250) and identified by MS-library, and quantification was performed through calibration curves of external standards.

### 2.7 Astaxanthin Content

Prior to the analysis of astaxanthin and astaxanthin esters, the lipids in the process waters were extracted with chloroform and methanol according to the method described by Bligh and Dyer^17^ with a reduced amount of solvent applied.^18^ The lipid extracts were evaporated to dryness under nitrogen and redissolved in 1 mL of heptane. The extracts (50 μL) were injected and analyzed on an HPLC (Agilent Technologies 1100; column: Kinetex 2.6u 100A, 100 × 4.6 mm, Phenomenex) using isocratic elution with heptane/acetone (86:14) at 1.2 mL/min. Astaxanthin and astaxanthin esters were detected at 470 nm and quantified against an external standard by using a single point calibration.

### 2.8 Element Content

Determination of nutritional (selenium (Se), zinc (Zn), copper (Cu), iron (Fe), manganese (Mn), chromium (Cr), calcium (Ca), potassium (K), phosphorus (P), magnesium (Mg), and sodium (Na)) and potentially toxic (arsenic (As), nickel (Ni), lead (Pb), mercury (Hg), and cadmium (Cd)) elements in the process waters was done using inductively coupled plasma MS (ICP-MS) (iCAPq, Thermo-Fischer, Germany) in KED mode (helium as cell gas) following digestion of the samples with concentrated nitric acid (SPS Science, France) using a microwave oven (Multiwave 3000, Anton Paar, Graz, Austria). Quantification was done using external calibration with standard solutions made from certified stock solutions (SPS Science, France) and using rhodium as an internal standard (SPS Science, France). A certified reference material, TORT-3 (lobster hepatopancreas) (NRCC, Ottawa, Canada), was analyzed (*n* = 7) together with the samples, and the obtained values were in good agreement with the certified reference values.

### 2.9 Storage Study of SBW and SPW

Five liters of SBW and SPW collected at the factory in February and March 2016 was stored at 4 °C for 18 days in a 12 L plastic bucket with the lid on and no stirring. To monitor the biochemical degradation, samples were taken daily and stored at −80 °C until analysis of lipid oxidation, total volatile basic nitrogen (TVB-N), volatile compounds, and odor.

#### 2.9.1 Lipid Oxidation

Measurement of malondialdehyde (MDA) was performed using DNPH derivatization and LC–MS following the method by Tullberg et al.^19^

#### 2.9.2 Volatile Compounds

Volatile compounds during storage were also measured in the stored waters as described in Section 2.6.

#### 2.9.3 TVB-N

Total volatile basis nitrogen (TVB-N) was measured according to the method described by Rawdkuen et al.^20^ Briefly, 4 mL of the sample was mixed with 6 mL of 4% trichloroacetic acid followed by vortexing for 1 min and centrifugation at 3000*g* for 15 min. Two milliliters of the supernatant was placed in the outer ring of a Conway cell and 2 mL of 1% boric acid was placed in the inner ring, and after closing the lid, the cell was incubated for 60 min at 37 °C. Thereafter, a known amount of 2 mM HCl was added to the inner ring until the color changed from green to pink. TVB-N was calculated based on the amount of HCl used.

#### 2.9.4 Sensory Analysis of Odor

Sensory analysis of odor was performed with five participants that were first subjected to a training session to agree on the most suitable attributes characterizing the SBW and SPW. These were ″boiled shrimp″, ″shellfish″, and ″fishiness″. During the storage at 4 °C, samples were daily smelled in E-flasks (80 mL in each) and the intensity of attributes was rated on a scale of 0–10.

#### 2.9.5 Microbiology Analyses

The presence of psychrotolerant bacteria and hydrogen sulfide producing and non-hydrogen sulfide producing bacteria was investigated according to the Nordic Committee on Food Analysis (NMKL) method 184.^21^ Psychrotolerant bacteria was determined using Long and Hammer agar media incubated at 15 °C for 5 days; hydrogen sulfide producing and non-hydrogen sulfide producing bacteria were determined upon culturing on iron agar containing 0.04% l-cysteine. Briefly, 0.5 mL of the sample was mixed with 4.5 mL of sterile 0.9% NaCl solution and vortexed. Thereafter, a dilution series of the stock was made and the appropriate dilution giving bacterial colonies of 30–300 was cultured on the plate.

### 2.10 Statistical Analysis

Statistical differences among sample means of analyses were studied by analysis of variance (ANOVA) at *p* ≤ 0.05 using MINITAB release 16. The values are reported as mean values ± SD. Analyses were performed in duplicates except for protein content and volatile compound measurements that were in triplicates.

## 3 Results and Discussion

### 3.1 Compositional Characteristics of SBW and SPW

pH, ionic strength, dry matter, total fatty acids, eicosapentaenoic acid (EPA), docosahexaenoic acid (DHA), protein, total essential amino acids (EAA), free amino acids, and astaxanthin for SBW are shown in Table 1, and the same parameters for SPW are reported in Table 2. EPA and DHA were reported only for SBW (Table 1). pH values of SBW varied from 8.5 to 9.0, and the ionic strength of SBW ranged from 0.24 to 0.58% (Table 1). pH of SPW varied from 8.1 to 8.7, while ionic strength varied from 0.009 to 0.13% (Table 2). The shrimp muscle pH was measured to be 7.1; the pH of SBW and SPW was most likely affected also by the pH of the tap water used for processing (pH ∼8.5) and by compounds like calcium carbonate leaching out from the shell into the water. Wet shrimp shells contain 4.4% calcium.^22^ Other studies reported pH of 6.4^2^ and 7.7^8^ in shrimp cooking and pH of 8.3 in shrimp peeling water.^23^

**Table 1:** Characterization of SBW Collected Oct 2015–Oct 2016 in Terms of pH, Ionic Strength, Dry Matter, Protein, Total Fatty Acid Content, EPA and DHA, Total Essential Amino Acids (EAA) (% of Total Amino Acids), Free Amino Acids (FAA) (% of Total AA), as well as Astaxanthin in Esterified and Free Form^a^

										astaxanthin	
month	pH	ionic strength (Na Cl (%))	dry matter (%)	total fatty acid (g/L)	EPA (g/L)	DHA (g/L)	protein (g/L)	total EAA (%)	FAA (%)	esterified (mg/L)	free (mg/L)
Oct 2015	8.51 ± 0.01^K^	0.31 ± 0.00^G^	3.68 ± 0.02^B^	2.19 ± 0.00^8^	0.25 ± 0.01D^E^	0.21 ± 0.01^B^	16.69 ± 0.58^B^	43.0 ± 0.4^A^	11.9 ± 0.7^ABC^	2.55 ± 0.22B^CDE^	<LOD
Nov 2015	8.90 ± 0.00^D^	0.51 ± 0.01^B^	3.33 ± 0.00^C^	2.90 ± 0.06^BC^	0.32 ± 0.01^CD^	0.22 ± 0.01^B^	15.12 ± 0.92^BCD^	45.0 ± 1.3^A^	10.4 ± 0.3^ABC^	2.81 ± 0.10B^CD^	<LOD
Dec 2015	8.76 ± 0.01^I^	0.34 ± 0.00^F^	3.10 ± 0.00^D^	3.91 ± 0.08^A^	0.17 ± 0.01^FG^	0.12 ± 0.00^B^	13.69 ± 0.78^D^	46.4 ± 1.5^A^	12.6 ± 2.0^ABC^	1.82 ± 0.53^EF^	<LOD
Jan 2016	8.88 ± 0.00^E^	0.43 ± 0.00^C^	3.02 ± 0.03^D^	1.71 ± 0.06^E^	0.26 ± 0.00D^E^	0.16 ± 0.01^B^	13.75 ± 0.31^D^	48.6 ± 1.0^A^	13.3 ± 0.9^A^	3.38 ± 0.07^AB^	<LOD
Feb 2016	8.65 ± 0.00^J^	0.31 ± 0.00^G^	2.99 ± 0.06^D^	1.44 ± 0.08^E^	0.22 ± 0.03^EF^	0.15 ± 0.01^B^	13.81 ± 0.25^D^	46.7 ± 3.5^A^	9.3 ± 0.5^ABC^	1.99 ± 0.45^DEF^	<LOD
Mar 2016	8.80 ± 0.00^H^	0.24 ± 0.00^J^	2.12 ± 0.01^E^	0.82 ± 0.06^F^	0.11 ± 0.00^G^	0.18 ± 0.08^B^	8.44 ± 0.42^E^	45.9 ± 1.4^A^	13.1 ± 0.7^AB^	1.54 ± 0.02^F^	<LOD
Apr 2016	8.86 ± 0.01^F^	0.38 ± 0.00^E^	3.35 ± 0.06^C^	2.05 ± 0.10^D^	0.29 ± 0.02^DE^	0.13 ± 0.01^B^	15.37 ± 0.55^BCD^	ns	ns	3.07 ± 0.18^ABC^	<LOD
May 2016	8.93 ± 0.01^C^	0.30 ± 0.00^H^	3.35 ± 0.01^C^	2.28 ± 0.06^D^	0.39 ± 0.01^BC^	0.16 ± 0.00^B^	16.00 ± 0.44^BC^	48.6 ± 0.6^A^	8.7 ± 0.7^BC^	3.48 ± 0.35^AB^	<LOD
Jun 2016	8.98 ± 0.01^B^	0.27 ± 0.00^I^	3.00 ± 0.01^D^	3.01 ± 0.15^B^	0.40 ± 0.02^B^	0.18 ± 0.02^B^	14.60 ± 0.61^CD^	47.6 ± 1.2^A^	9.7 ± 1.1^ABC^	3.51 ± 0.17^AB^	<LOD
Jul 2016	8.85 ± 0.01^F^	0.35 ± 0.00^F^	3.04 ± 0.00^D^	2.10 ± 0.01^D^	0.25 ± 0.01^DE^	0.15 ± 0.01^B^	13.98 ± 0.37^D^	45.2 ± 3.0^A^	8.5 ± 0.6^BC^	3.50 ± 0.14^AB^	<LOD
Aug 2016	9.01 ± 0.01^A^	0.59 ± 0.00^A^	3.52 ± 0.10^B^	2.16 ± 0.02^D^	0.30 ± 0.01^DE^	0.16 ± 0.00^B^	16.27 ± 0.68^BC^	45.0 ± 1.6^A^	11.3 ± 1.9^ABC^	2.26 ± 0.08^CDEF^	<LOD
Sep 2016	8.82 ± 0.01^G^	0.39 ± 0.00^D^	4.31 ± 0.03^A^	2.71 ± 0.08^C^	0.62 ± 0.05^A^	0.45 ± 0.02^A^	20.85 ± 0.55^A^	46.1 ± 0.8^A^	10.0 ± 0.8^ABC^	3.83 ± 0.12^A^	<LOD
Oct 2016	8.94 ± 0.01^C^	0.28 ± 0.00^I^	3.00 ± 0.00^D^	2.27 ± 0.05^D^	0.30 ± 0.01^DE^	0.19 ± 0.00^B^	14.83 ± 1.04^CD^	45.2 ± 2.8^A^	8.4 ± 1.7^C^	2.60 ± 0.05^BCDE^	<LOD
median	8.8	0.34	3.0	2.19	0.29	0.16	14.8	46.0	10.2	2.81	

a Data are given as average value ± SD (*n* = 2 for dry matter, fatty acid analyses, amino acid, and astaxanthin analyses and *n* = 3 for the rest of analyses). Data within the same column carrying different letters are significantly different on a *p* < 0.05 level. ns = no sample.

**Table 2:** Characterization of SPW Oct 2015–Oct 2016 in Terms of pH, Ionic Strength, Dry Matter, Protein, Total Fatty Acid Content, Total Essential Amino Acids (EAA) (% of Total Amino Acids), Free Amino Acids (FAA) (% of Total AA), as well as Astaxanthin in Esterified and Free Form^a^

								astaxanthin	
month	pH	ionic strength (Na Cl (%))	dry matter (%)	total fatty acid (g/L)	protein (g/L)	total EAA (%)	FAA (%)	esterified (mg/L)	free (mg/L)
Oct 2015	8.13 ± 0.02^I^	0.01 ± 0.00^G^	0.22 ± 0.01^FGH^	0.21 ± 0.04^BC^	0.75 ± 0.05^GH^	42.9 ± 0.0^B^	24.8 ± 2.7^AB^	1.06 ± 0.06^AB^	<LOD
Nov 2015	8.73 ± 0.01^A^	0.13 ± 0.00^A^	0.91 ± 0.01^A^	0.59 ± 0.20^A^	3.60 ± 0.16^A^	46.6 ± 1.0^AB^	16.3 ± 1.0^AB^	1.67 ± 0.05^A^	<LOD
Dec 2015	8.12 ± 0.01^I^	0.01 ± 0.00^G^	0.21 ± 0.00^GH^	0.14 ± 0.04^BC^	0.64 ± 0.03^H^	45.6 ± 1.8^AB^	26.2 ± 5.4^A^	0.91 ± 0.24^AB^	<LOD
Jan 2016	8.31 ± 0.02^F^	0.02 ± 0.00^B^	0.26 ± 0.00^EF^	0.16 ± 0.00^BC^	1.16 ± 0.07^DE^	46.5 ± 1.8^AB^	19.0 ± 6.9^AB^	0.91 ± 0.42^AB^	<LOD
Feb 2016	8.13 ± 0.01^HI^	0.02 ± 0.00^C^	0.29 ± 0.00^DE^	0.13 ± 0.01^BC^	1.05 ± 0.06^EF^	45.4 ± 1.3^AB^	18.6 ± 2.7^AB^	1.31 ± 0.02^AB^	<LOD
Mar 2016	8.12 ± 0.01^I^	0.01 ± 0.00^F^	0.19 ± 0.01^H^	0.07 ± 0.00^C^	0.67 ± 0.03^H^	46.1 ± 1.3^AB^	23.8 ± 0.2^AB^	1.30 ± 0.11^AB^	<LOD
Apr 2016	8.45 ± 0.01^C^	0.01 ± 0.00^F^	0.22 ± 0.01^FGH^	0.20 ± 0.00^BC^	0.89 ± 0.03^FG^	49.6 ± 1.1^A^	16.7 ± 0.6^AB^	1.17 ± 0.18^AB^	<LOD
May 2016	8.57 ± 0.01^B^	0.02 ± 0.00C^D^	0.34 ± 0.02^C^	0.32 ± 0.04^B^	1.65 ± 0.07^C^	47.9 ± 0.0^AB^	18.9 ± 0.2^AB^	0.80 ± 0.42^B^	<LOD
Jun 2016	8.31 ± 0.01^F^	0.02 ± 0.00^E^	0.42 ± 0.02^B^	0.57 ± 0.06^A^	2.34 ± 0.03^B^	43.6 ± 3.3^B^	13.7 ± 0.9^B^	1.20 ± 0.13^AB^	<LOD
Jul 2016	8.42 ± 0.01^D^	0.01 ± 0.00^G^	0.28 ± 0.01^DE^	0.33 ± 0.00^B^	1.29 ± 0.02^D^	44.8 ± 0.7^AB^	16.1 ± 2.0^AB^	1.19 ± 0.10^AB^	<LOD
Aug 2016	8.16 ± 0.01^H^	0.01 ± 0.00^G^	0.22 ± 0.01^FGH^	0.26 ± 0.00B^C^	0.89 ± 0.08^FG^	44.6 ± 0.2^AB^	19.9 ± 1.5^AB^	0.64 ± 0.16^B^	<LOD
Sep 2016	8.38 ± 0.01^E^	0.02 ± 0.00^DE^	0.32 ± 0.01^CD^	0.26 ± 0.01^BC^	1.51 ± 0.10^C^	46.9 ± 0.8^AB^	17.9 ± 3.0^AB^	0.89 ± 0.29^AB^	<LOD
Oct 2016	8.28 ± 0.01^G^	0.01 ± 0.00^F^	0.23 ± 0.01^FG^	0.21 ± 0.00^BC^	0.96 ± 0.03^EF^	45.5 ± 2.5^AB^	18.2 ± 2.5^AB^	1.23 ± 0.02^AB^	<LOD
median	8.31	0.01	0.31	0.21	1.05	45.5	18.4	1.17	

a Data are given as average value ± SD (*n* = 2 for dry matter, fatty acid analyses, amino acid, and astaxanthin analyses and *n* = 3 for the rest of analyses). Data within the same column carrying different letters are significantly different on a *p* < 0.05 level.

The dry matter of SBW and SPW was 2.1–4.3% and 0.18–0.90%, respectively (Tables 1 and 2), showing that SPW was around 10-fold more diluted than SBW. In the present study, the amount of water that had been used per ton of shrimp was 0.66 m^3^ at the stage where SBW was sampled, while it was 19 times higher at the SPW sampling point. However, the dry matter of both types of waters differed by a factor that was lower than 19, reflecting the continuous leaching of components during peeling and transportation.

The median value for protein was 14.8 g/L, and 86% of the SBW specimens showed a protein content above 13.6 g/L (Table 1). SPW showed 6- to 14-fold lower protein content compared to SBW, and it contained 0.6–3.6 g protein/L. Total nitrogen contents of shrimp boiling waters studied in France and Spain were 1.49 and 3.0 g/L, respectively, equal to 8.3 and 16.7 g/L of crude protein using a nitrogen conversion factor of 5.58.^24^ Thus, the values were similar to ours. Reported values for protein and nitrogen content of peeling waters from shrimp processing in Denmark and Brazil were 0.19 and 0.9 g/L, respectively, thus also in agreement with our data.^7,23^

**Figure 1:**
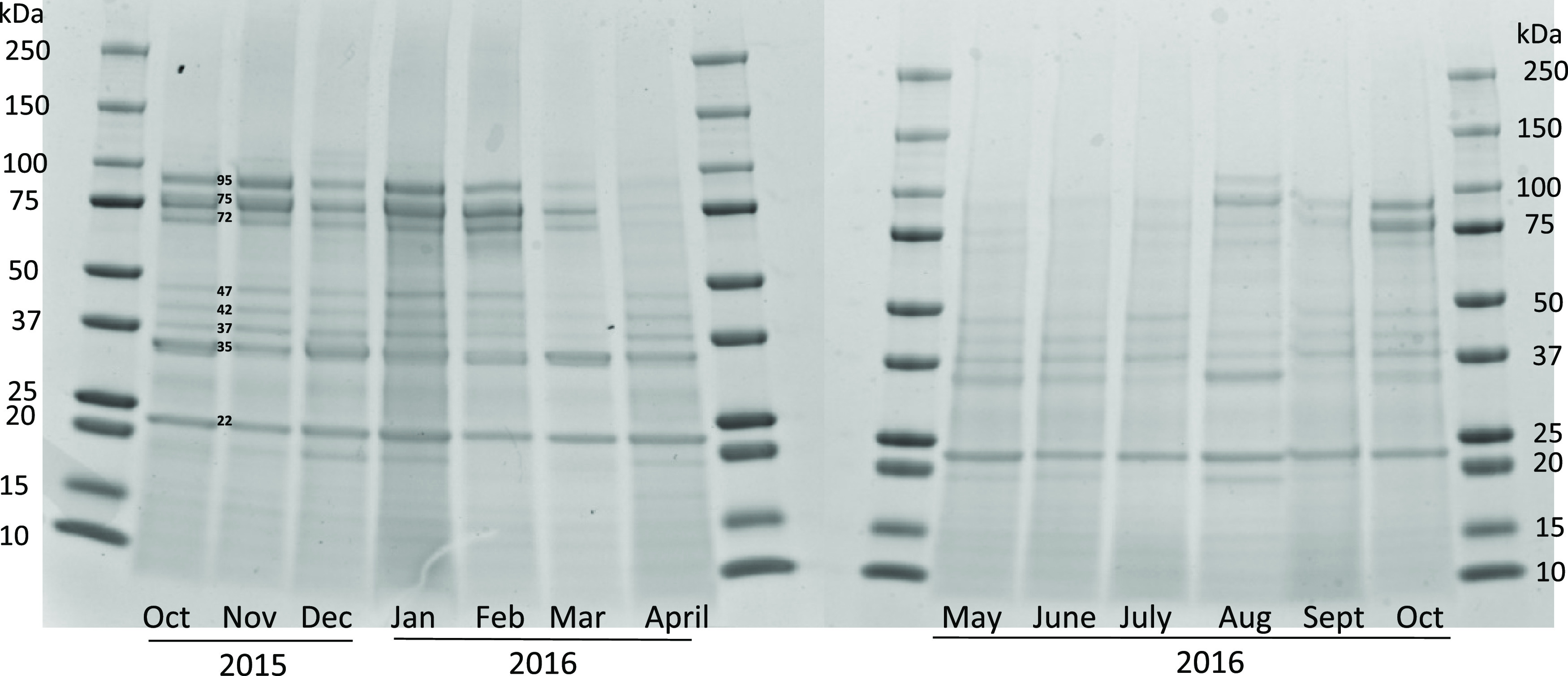
Polypeptide profiling of SBW over the 13-month sampling. Electrophoresis was carried out using Mini-protean TGX 4–20% pre-cast gels (Bio-Rad Laboratories, USA). Protein bands were stained by Coomassie Brilliant blue G-250. Each well was loaded with 20 μg protein.

Polypeptides leached from *P. borealis* during processing had a wide range of molecular weights (Figure 1). Clear bands were present at 22, 35, 37, 42, and 47 kDa across all SBWs; however, bands at 72 and 75 kDa were also seen in some of the SBW specimens (October 2015–March 2016). The bands at 42 and 37 kDa were tentatively identified as actin and β-tropomyosin, respectively.^25,26^ The steady band at 95 kDa was assumed to be paramyosin,^27^ which was present in all samples, although the intensity was lower in April–July samples. The latter could be due to higher protease activities in these months, although there was no visible accumulation of smaller peptides. It is however possible these were too small to remain on the gel. Martinez et al.^28^ reported on a series of bands above 70 to 100 kDa that gave a positive interaction with the anti-myosin antiserum when the polypeptide profile of *P. borealis*, *Penaeus japonicus*, and *Penaeus monodon* was investigated, giving more indication of the nature of bands at 72 and 75 kDa. The 22 kDa band is tentatively identified as myosin light chain,^29^ which is dominant in all SBW samples.

The relative content of free amino acids (FAA), as a marker of proteolysis, was quantified in SBW and SPW (Tables 1 and 2). The percentage of FAA based on total amino acids was significantly (*p* < 0.05) higher in SPW than SBW. For both waters, the content of FAA varied over the sampling period between 8.4 and 13.3% (median: 10.2%) for SBW and between 16.1 and 26.2% (median: 18.4%) for SPW, with no clear pattern linked to sampling month; a higher fluctuation in FAA was observed for SPW than SBW. It has been described that the enzymatic process takes place during the transportation and maturation of shrimps before the boiling.^23^ Enzymes are then assumed to be inactivated by the steaming process. Cambero et al.^30^ observed a correlation between FAA in shrimp broth and shrimp cooking temperature, where a lower FAA was observed when cooking at 95–100 °C than ≤85 °C. It seems likely that the differences in post-harvest storage time prior to processing of the shrimps, as well as maturation time, largely affected the level of FAA in both water types, but more so in SPW as this water was incubated for a longer period with the shrimps and therefore allowed a more extensive leaching of soluble compounds as FAA. The concentration and type of FAA were previously reported to be a decisive factor for the sensory profile of shrimp cooking juice.^30^

From the amino acid determination, it was shown that the essential amino acids (EAA) counted for around 45% of total amino acids for both SBW (median: 46.0%) and SPW (median: 45.5%) even though the absolute content of amino acids was much higher in SBW than SPW. These numbers were comparable with those reported for fish meal^31^ and for shrimp head protein hydrolysate (0.46),^32^ and the shrimp process waters thus have potential as a highly nutritional feed and food source provided that the *proteinaceous* compounds are recovered. Others have reported that 37% of the total amino acids in shrimp cooking juice were EAA,^33^ and in an extract containing odor-active compounds from roasted shrimp, 19% were EAA.^34^

The total fatty acid content of SBW ranged from 1.4 to 3.9 g/L (median value 2.2), with minimum and maximum values belonging to March and December samples, respectively (Table 1). The fatty acid content in SPW varied between 0.07 and 0.59 g/L (median value 0.21) (Table 2). Total lipid in *P. borealis* muscle and cephalothorax (i.e., the head and the thorax) was earlier reported to be 0.98 and 4.98%, respectively,^35^ explaining the origin of the fatty acids in SBW and SPW, which during processing were in contact with both whole peel-on shrimps and peeled shrimps. Eicosapentaenoic acid (C20:5 n-3, EPA) and docosahexaenoic acid (C22:6 n-3, DHA) ranged from 0.17 to 0.62 g/L and 0.12 to 0.45 g/L in SBW with median values being 0.29 and 0.16, respectively. EPA and DHA accounted for 4.2–22.8% and 3.0–22.4% of total fatty acids, respectively. Generally, there were higher levels of EPA than DHA, and in 90% of the SBW samples, the relative amount of EPA was over 13%, while 76% of the SBW samples had below 10% DHA. This reflects the higher content of EPA in comparison to DHA that has been reported for whole *P. borealis* as well as its muscle and cephalothorax: 12.5 vs 7.7%, 23.1 vs 18.9%, as well as 13.1 vs 10.9%, respectively.^35,36^ As mentioned earlier, the volume of SPW to process 1 kg fresh shrimp was 19-fold higher than that of SBW; however, the fact that the total fatty acid content in 12 of 13 SPW specimens was ≤11-fold lower than in SBW indicates that peeling and transportation steps were also effective in leaching out lipids from the shrimps.

Astaxanthin is the predominant carotenoid present in shrimp, and our data revealed that the majority of the astaxanthin leaching out into process waters was in the form of astaxanthin ester (Tables 1 and 2). There was however no clear pattern in the astaxanthin concentration present in waters during the sampling period. In SBW, astaxanthin ester content varied from 1.8 to 3.8 mg/kg (median: 2.8 mg/L), while there was only 0.04–0.25 mg/L (median: 0.11 mg/L) free astaxanthin (Table 1). In SPW, corresponding numbers were 0.6–1.7 mg/L (median: 1.2 mg/kg) and 0–0.27 mg/L (median: 0.01 mg/L). These concentrations were lower than those earlier reported in shrimp (*Penaeus vannamei*) cooking wastewater, 10–13 mg/L.^2^ Based on median numbers, the contents of astaxanthin ester and free astaxanthin were thus diluted only 2.3- and 5.7-fold, respectively, between the sampling points for SBW and SPW, indicating a relatively larger leaking during transportation and peeling of shrimps than during boiling.

### 3.2 Content of Nutritional and Potentially Toxic Elements in SBW and SPW

Several studies have shown that *P. borealis* is a good source of nutritional elements.^37,38^Table 3 shows that such elements, which in general are water-soluble, are leaching out to SBW and SPW during shrimp processing. The lower levels in SPW compared to SBW reflect the severe dilution during transport and peeling. In SBW, the five most enriched elements were Na > K > P > Ca > Mg. For SPW, corresponding data were Na > Ca = K > P > Mg. In SBW, 4.7 and 4.67 mg Zn and Cu/L were also found. As a comparison, the amounts of Na, K, P, Ca, and Mg in peeled *P. borealis* were reported to be 2361, 2014, 19,623, 166,843, and 8112 mg/kg, respectively, while Zn, CU, Fe, and Mn levels were 15.9, 3.9, 53.6, and 11.1 mg/kg, respectively.^37^

**Table 3:** Concentration of Nutritional Elements in SBW and SPW (Median and Concentration Range during the 13 Months, *N* = 1)^a^

	SBW		SPW	
element	median	range	median	range
Se	0.16	0.09–0.21	0.05	0.02–0.09
Zn	4.67	<3.1–7.95	<3.1	<3.1–3.55
Cu	4.71	2.36–7.80	<0.7	<0.7–3.01
Fe	<3.5	<3.5–7.19	<3.5	<3.5–5.73
Mn	0.10	0.05–0.25	<0.03	<0.03–0.12
Cr	<0.06	<0.06–<0.06	<0.06	<0.06–<0.06
Ca	152	71–260	44	34–92
K	702	573–1122	44	34–186
P	361	195–527	31	20–119
Mg	36	22–58	8.7	7.0–14
Na	1423	871–3025	100	69–656

a All concentrations are in mg/L.

Table 4 shows the results from the analysis of toxic elements in SBW and SPW samples. All levels for Ni, Pb, Hg, and Cd were below the limit of detection (LOD) of the ICP-MS method used. These findings are in agreement with a recent study on the muscle tissue of *P. borealis*, where low levels for Pb (0.0005 ± 0.002 mg/kg), Hg (0.020 ± 0.009 mg/kg), and Cd (0.129 ±0.038 mg/kg) were reported.^35^ In contrast, higher levels of As were found in the SBW and SPW samples, reflecting the relatively high As levels earlier reported for shrimps (*P. borealis*; ≤96 mg/kg).^39^ However, the nontoxic and water-soluble arsenobetaine has been described as the predominant arsenic compound in *P. borealis*,^35^ and it is therefore likely that it is this form that is recovered in the SBW and SPW. The levels of toxic elements reported here do not pose specific food safety concerns.^40^ For both nutritional and potentially toxic elements, no systematic patterns related to season were observed, and hence, the results over the whole period are presented as median and range values.

**Table 4:** Concentration of Toxic Elements in SBW and SPW (Median and Concentration Range during the 13 Months, *N* = 1)^a^

	SBW		SPW	
element	median	range	median	range
As	4.88	3.45–7.32	0.34	0.22–1.25
Ni	<0.11	<0.11–<0.11	<0.11	<0.11–0.59
Pb	<0.02	<0.02–1.53	<0.03	<0.03–0.13
Hg	<0.02	<0.02–1.53	<0.02	<0.02–<0.02
Cd	<0.003	<0.003–<0.003	0.003	<0.003–0.02

a All concentrations are in mg/L.

### 3.3 Volatile Compounds in SPW and SBW

Several volatile compounds were detected and quantified in the process waters: butanal, 2-butanone, 1-penten-3-one, pentanal, 1-penten-3-ol, octane, 1-methyl pyridine, 3-methyl-1-butanol, 2-methyl-1-butanol, hexanal, 2-hexenal, heptanal, 2,5-dimethyl pyrizine, dl-limonene, benzaldehyde, 2,4-heptadienal, 2-methyl benzaldehyde, 2,6-nonadienal, dimethyl-1-dodecanamine, indole, and pristane. The concentration of volatiles in SPW fluctuated over the season, and most volatiles were present in low concentrations with median values <5 ng/g SPW. Exceptions were butanal, 2-butanone, 1-penten-3-ol, and pristane with medians of 6, 111, 6, and 31 ng/g SPW. The concentration of volatiles in SBW was much higher and had large fluctuations over the sampling period. The following volatiles were quantified in concentrations > 25 ng/g (median): butanal (32 ng/g), 2-butanone (141 ng/g), 1-penten-3-ol (30 ng/g), 3-methyl-1-butanol (30 ng/g), 2,5-dimethyl pyrazine (34 ng/g), 2,4-heptadienal (36 ng/g), and pristane (43 ng/g). The following volatiles were quantified in concentrations < 5 ng/g (median) in SBW: 1-penten-3-one, dl-limonene, 2-methyl benzaldehyde, and dimethyl-1-dodecanamine.

**Figure 2:**
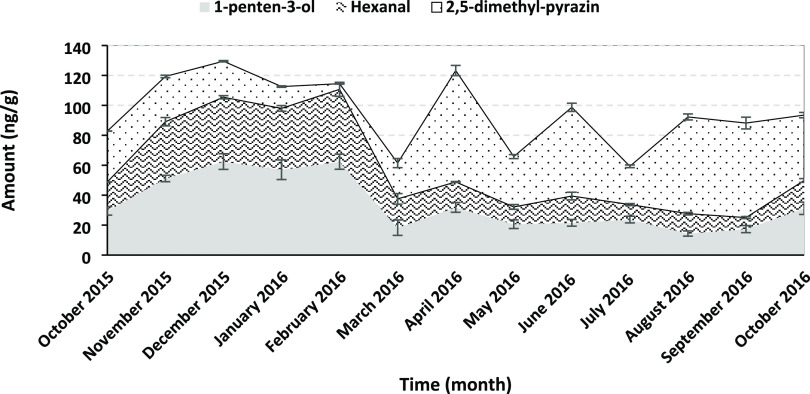
Concentration of volatiles from SBW sampled over a period of 13 months detected by GC–MS and quantified using external standards. (A) 1-Penten-3-ol, (B) hexanal, and (C) 2,5-dimethyl pyrazine. Data points show average ± SD; *n* = 3.

The content of 1-penten-3-ol, hexanal, and 2,5-dimethylpyrazine in SBW is presented in Figure 2. 1-Penten-3-ol and hexanal are derived from the lipid oxidation of n-3 and n-6 PUFA, respectively. These two volatiles were significantly more concentrated in the winter months (Nov–Feb) than in the other months. 2,5-Dimethylpyrazine is formed in foods during cooking or roasting processes due to the Maillard reaction between sugars and proteins. This volatile is responsible for the roasted and nutty aroma extracted from roasted shrimps.^34^ In SBW, there was a tendency toward higher concentration in the winter months regarding the lipid oxidation-derived volatiles. For instance, butanal (significantly higher in Nov–Feb), 2-butanone (significantly (*p* < 0.05) higher in Dec), 1-penten-3-on, pentanal, 1-penten-3-ol (significantly (*p* < 0.05) higher in Nov–Feb; Figure 2), hexanal (Figure 2), 2-hexenal (significantly (*p* < 0.05) higher in Oct 15, Jan–Feb), heptanal (higher in Feb, not significant in many other months), 2,4-heptadienal (significantly higher in Dec (*p* < 0.05), not significant from Oct to Jan), and 2,6-nonadienal (significantly (*p* < 0.05) higher in Dec, but not from Jan to Feb). However, the total fatty acid content of SBW was significantly (*p* < 0.05) higher in December than in the other sampling months (Table 1); this was not the case for November, January, and February. Also, there were the same levels of the antioxidant astaxanthin in this period. Thus, the reason for the higher amount of volatile lipid oxidation compounds in SBW in winter months would need to be evaluated further. The higher concentration of 2,5-dimethyl pyrazine in SBW compared with SPW could be due to the dilution between the two sampling points and the fact that there is no de novo formation of this volatile compound after the steaming step.

Limited studies have been reported on volatiles quantified in shrimp process waters. Besides the characterization of 2,5-dimethyl pyrazine as an odor-active compound formed due to roasting,^34^ one study evaluated the major flavor compounds of shrimp cooking juice (Jarrault et al.^8^) and another study investigated the odor-active compounds extracted from roasted shrimps.^34^ In the former study, five compounds played a major role for the natural shrimp flavor of cooking juice: benzealdehyde, 1-octen-3-ol, 2,3,5-trimethyl pyrazine, 3-ethyl-2,5-dimethylpyrazine, and decanal.^8^ In our study, the benzaldehyde concentration in SBW fluctuated over the sampling period (median: 15 ng/g) and 1-octen-3-ol could not be quantified as it co-eluted with other compounds.

Depending on the application of SBW and SPW, their volatile profile may change further during downstream processing steps such as separation, condensation, or drying, all which may induce, e.g., oxidation and interaction with other compounds.

Overall, it is assumed that the variation in levels and types of nutrients and potential toxic elements leached into SBW and SPW is a cumulative effect of several parameters such as the length of the postmortem storage of shrimps prior to processing, the exact biochemical profile of the shrimps, and the mechanical forces during shrimps processing. Indeed, there may have been certain seasonality in the composition of the shrimps, but we believe that the impact from other factors many times overshadowed these effects. Nevertheless, the 13 samples taken gives a solid insight into the span of variation to be expected during a full-year cycle in a shrimp processing factory.

### 3.4 Storage Ability of SBW and SPW under Cold Conditions

**Figure 3:**
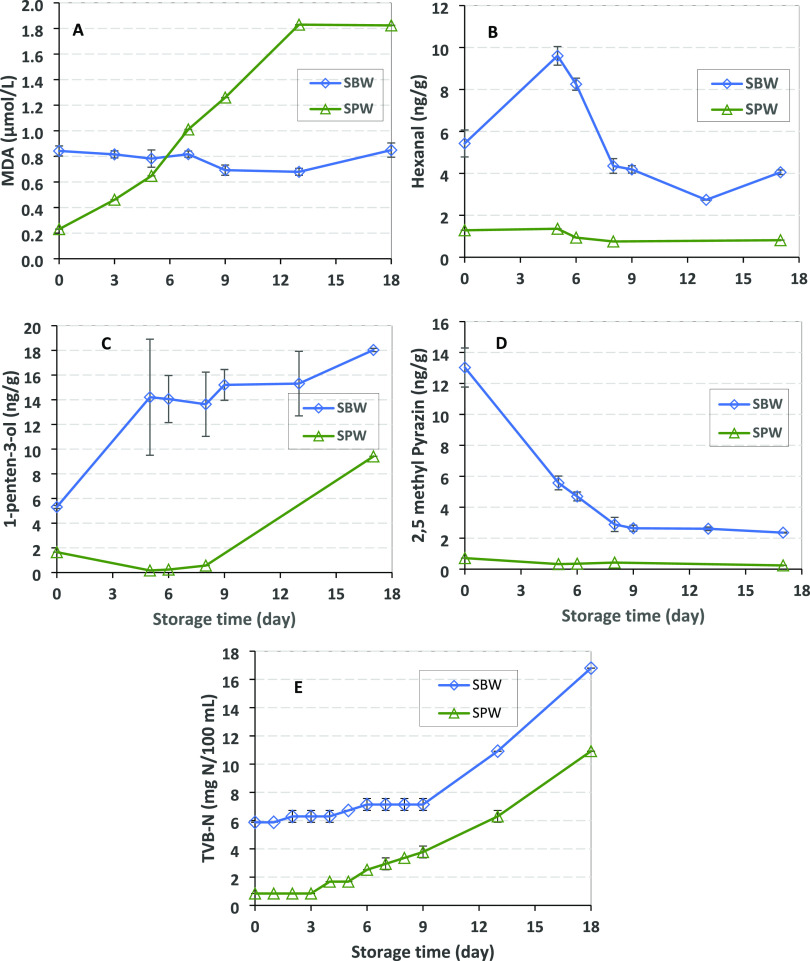
Characteristics of SPW and SBW during cold storage (4 °C). (A) MDA, (B) hexanal, (C) 1-penten-3-ol, (D) 2,5-dimethyl pyrazine, and (E) TVB-N. Data points show average ± SD; *n* = 2.

During storage of SBW and SPW, pH, lipid oxidation (MDA, volatile compounds), TVB-N, and odor were monitored. The pH of SPW decreased gradually during storage until day 13 when it reached 7.2 and 7.6, with minor changes thereafter. For SBW samples, pH fluctuated until day 9 and then slightly decreased until day 18 (data not shown). MDA showed a slight increase over time in SPW (0.2–1.0 μmol/L) but leveled out after day 13. In SBW, MDA fluctuated from 0.69 to 0.84 μmol/L during 18 days of storage (Figure 3A).

The same volatiles were quantified during cold storage of the process waters as for the 13 month sampling period. The profile of butanal, heptanal, pentanal and 2,4-heptadienal, benzaldehyde 1-penten-3-on, benzaldehyde, and 3-methyl butanol measured in SBW over the storage period is shown in Figure S1, and the profile of 1-penten-3-ol, hexanal, and 2,5-dimethyl pyrazine is presented in Figure 3. Again, volatiles were present in lower concentrations in SPW than SBW at time 0, and the concentrations of several volatiles in SPW were <5 ng/g (below LOD). In SBW, aldehydes including some derived from lipid oxidation (butanal, heptanal, pentanal, and 2,4-heptadienal) slightly increased until day 6, after which the concentration decreased (butanal, pentanal, hexanal (Figure 3B), 2,4-heptadienal, and benzaldehyde). For heptanal, the concentration decreased already from the beginning. In SBW, hexanal had a significantly higher concentration at days 5 and 6 than the other storage days. Other volatiles generated from lipid oxidation such as 1-penten-3-on were low in concentration and without storage-induced changes, and 1-penten-3-ol (Figure 3C) increased in concentration during storage for SBW. The volatile 2,5-dimethyl pyrazine, which was discussed in relation to its roasted and nutty odor,^34^ decreased significantly during storage above in SBW (Figure 3D). The concentration of 3-methyl butanol was stable during storage of SBW.

In SPW, butanal, pentanal, hexanal, 2,4-heptadienal, benzaldehyde, and 1-penten-3-on were in concentrations below the LOD. The concentration of 1-penten-3-ol was as for SBW increasing during storage (Figure 3D). In addition, the volatile 2,5-dimethyl pyrazine was more or less absent in SPW (Figure 3E). For SPW, the concentration of 3-methyl butanol initially increased and then leveled out after 6 days of storage. The increase in concentration of 3-methyl butanol occurred earlier in SPW than SBW (Figure S1). 3-Methyl butanol has been reported as a useful freshness indicator in poultry associated with microbial growth.^41^ Hence, the different pattern for SBW and SPW for this volatile could be due to the differences in bacterial growth. A screening of the latter revealed that SPW contained a higher bacterial load at day 0, and that presence of hydrogen sulfide producing bacteria increased over time compared to SBW (Figure S2).

Waters were evaluated over time with respect to the odor attributes boiled shrimp, shellfish, and fishiness (Figure S2). The former was however completely absent in SPW, and the latter was absent in SBW. In SBW, boiled shrimp and shellfish odors were unchanged during the first 9 days, but in the storage period of 9–18 days, they were reduced from intensities of 75 and 40, respectively, to <20. Regarding SPW, the shellfish attribute had completely disappeared at day 8 and fishiness had increased to 80. At this time point, the storage of SPW was stopped as samples smelled putrid.

The sensory evaluation corresponded with the microbial load screening (Figure S3), which increased more in SPW than in SBW, and also comprised the growth of hydrogen sulfide producing bacteria, which were absent in SBW. For the psychrotolerant bacteria, the microbial load in SPW was more than double that of SBW at day 0 (4.2 vs 2 log CFU/mL) (Figure S3), which could be due to the longer processing time and increased contact of water with shrimps and equipment. The number of psychrotolerant bacteria increased in both process waters during storage. In SPW, it reached 8.1 log CFU/mL already at day 13 as compared to 5 log CFU/mL. In SPW, hydrogen sulfide producing bacteria grew gradually until day 7, reaching 7.3 log CFU/mL, which was stable toward the end of storage. However, non-hydrogen sulfide producing bacteria increased gradually until day 18. In SBW, hydrogen sulfide producing bacteria did not grow, but non-hydrogen sulfide producing bacteria increased gradually from days 2 to 18, rising from 2 log CFU/mL at day 0 to 7.5 log CFU/mL at day 18 (Figure S3). The hydrogen sulfide producing bacteria measurement comprises specific spoilage organisms common in chilled stored fresh fish and shellfish, e.g., *Shexanella* spp., *Aeromonas* spp., and Vibrionaceae.^21^ The sulfide odor from such bacteria could have a potential impact on the smell of the waters.

Earlier studies have documented the antibacterial activity of astaxanthin against *Listeria monocytogenes* and Enterobacteriaceae^42^ why it is possible that the higher astaxanthin level in SBW compared to SPW could be the reason for its better quality during cold storage, also with respect to MDA development.

For SBW, TVB-N values started off higher than for SPW (at 5.8 mg vs 0.8 N/100 mL) but were stable until day 9 (7.1 mg N/100 mL). In SBW, 10.9 mg N/100 mL was reached at day 13 (Figure 3E). In SPW, TVB-N values increased after day 3 and reached 1.6 mg N/100 mL, and then gradually increased and reached 10.9 mg N/100 mL at day 18. The TVB-N kinetics thus reflected the higher microbial stability of SBW than SPW.

## 4 Final Remarks

Compositional mapping of two process waters generated during shrimp processing, SBW and SPW, revealed that the former was richer, with up to 14.8, 3.9, and 3.8 g/L protein, fatty acids, and esterified astaxanthin, respectively. The relative amounts of EAA and LC n-3 PUFA (EPA and DHA) reached up to 49 and 39% of the total amino acids and fatty acids, respectively. Among nutritious elements, the highest levels were found for Na, K, P, Ca, and Mg. Toxic elements were below LOD except for As. In the volatile profile, both lipid oxidation- and Maillard reaction-derived compounds were found, the former particularly in the winter months. Apart from this, there were no systematic variations in the composition of SBW and SPW that were linked with season. Thus, the profile of the 13 samplings was most likely affected more by the pre-processing storage time and the processing *per se* compared to the actual sampling month. Storage stability was higher for SBW than SPW, shown, e.g., as lower levels of MDA, hexanal, and odor. The SPW compositional data, along with the SPW volumes generated, show that 70, 14, 0.076, and 10 kg of protein, fatty acids, astaxanthin, and phosphorous, respectively, are lost into this combined water stream per tonne of boiled and peeled shrimp. These findings can thus guide processing companies toward the best possible approaches to recover lost nutrients. For instance, since SBW contained significant amounts of proteins/polypeptides with sizes up to 75 kDa, flocculation followed by flotation could be a potential strategy to recover a protein-enriched biomass into which fatty acids and astaxanthin are also likely to partition. For the smaller peptides and free amino acids, filtration may be the most appropriate approach, while the remaining dissolved micronutrients could also be used as feed stock for, e.g., algae or fungi to produce new biomasses. Indeed, conversion of the complete waters to shrimp broth or flavor agents using, e.g., vacuum evaporation is another potential strategy. Even with fast cooling, results from volatile compounds and microbiology of this study however revealed that SPW should be subjected to potential value-adding as soon as possible after its generation, preferably within 3 days. SBW was more robust and could be pre-stored up to 9 days. Overall, the results indicate that there are great incentives in converting the lost shrimp-derived nutrients and some of its volatiles to products provided that cost-effective techniques are applied.

## Supporting Information Available

Volatile compounds measured; analyses of psychrotolerant bacteria and hydrogen sulfide and non-hydrogen sulfide producing bacteria in SBW and SPW; and sensory characteristics of SBW and SPW (PDF)
