# Supplementary material for: Biochemical Characterization and Storage Stability of Process Waters from Industrial Shrimp Production
Source: ACS Omega. 2021 Nov 10;6(46):30960–70. doi: 10.1021/acsomega.1c03304 (PMC8619652; doi:10.1021/acsomega.1c03304)
Supplement: Supplementary file 1 — ao1c03304_si_001.pdf [file ao1c03304_si_001.pdf]

Biochemical characterization and storage stability of process waters from industrial shrimp production

Bita Forghani[1*], Ann-Dorit Moltke Sørensen[2], Gustaf Fredeus[1], Kenneth Skaaning[2], Johan Johannesson[3], Jens J. Sloth[2], and Ingrid Undeland[1]

[1]*Food and Nutrition Science, Biology and Biological engineering, Chalmers University of Technology, Gothenburg, Sweden*
[2]National Food Institute, Technical University of Denmark, Kgs. Lyngby, Denmark
[3]*Räkor & Laxgrossisten AB, Gothenburg*

Corresponding author: Bita Forghani bita.forghani@chalmers.se

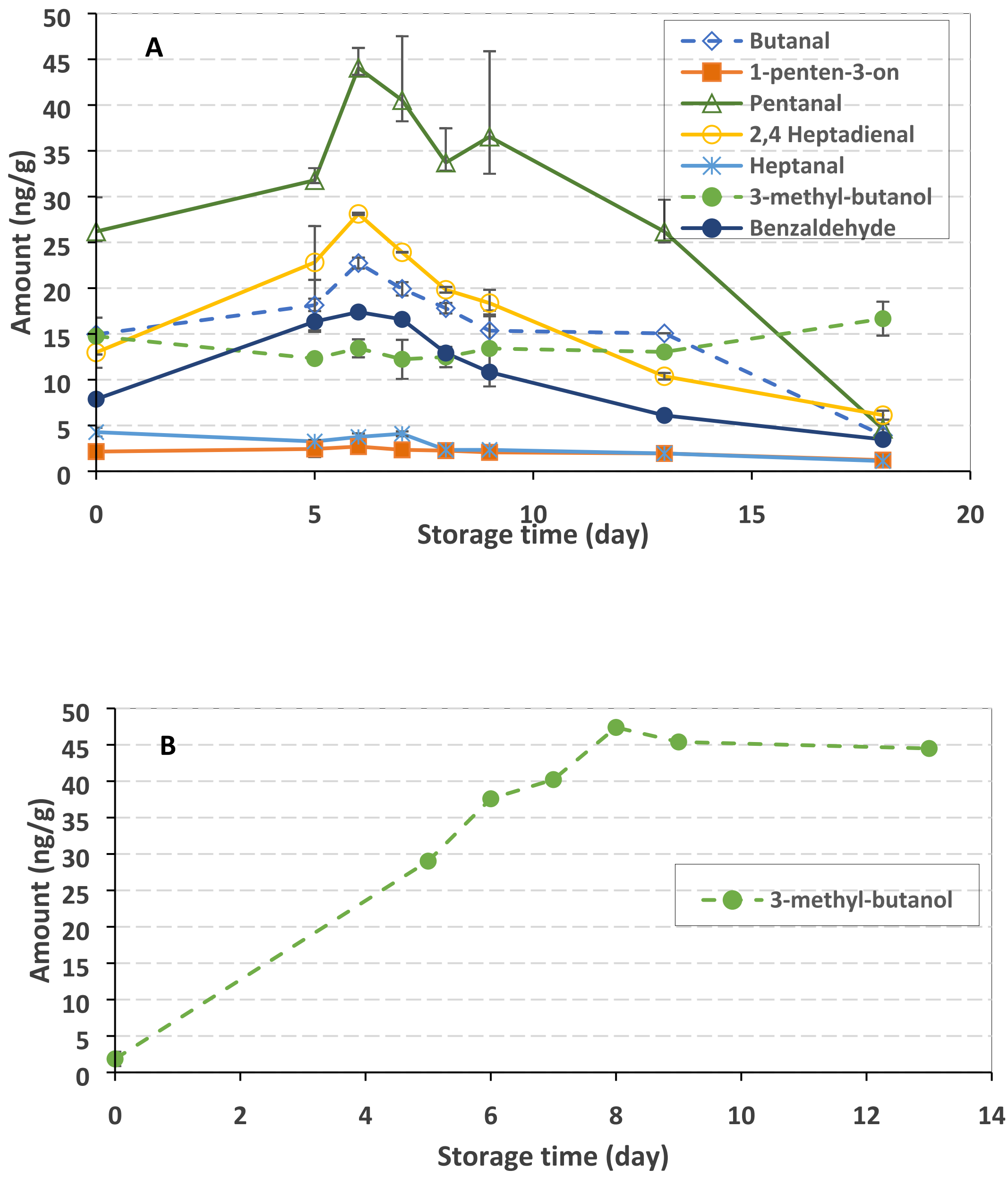

Figure 1S. Volatile compound(s) measured in SBW (A) and SPW (B) during cold storage (4°C). Data points show average ±SD, n=2.

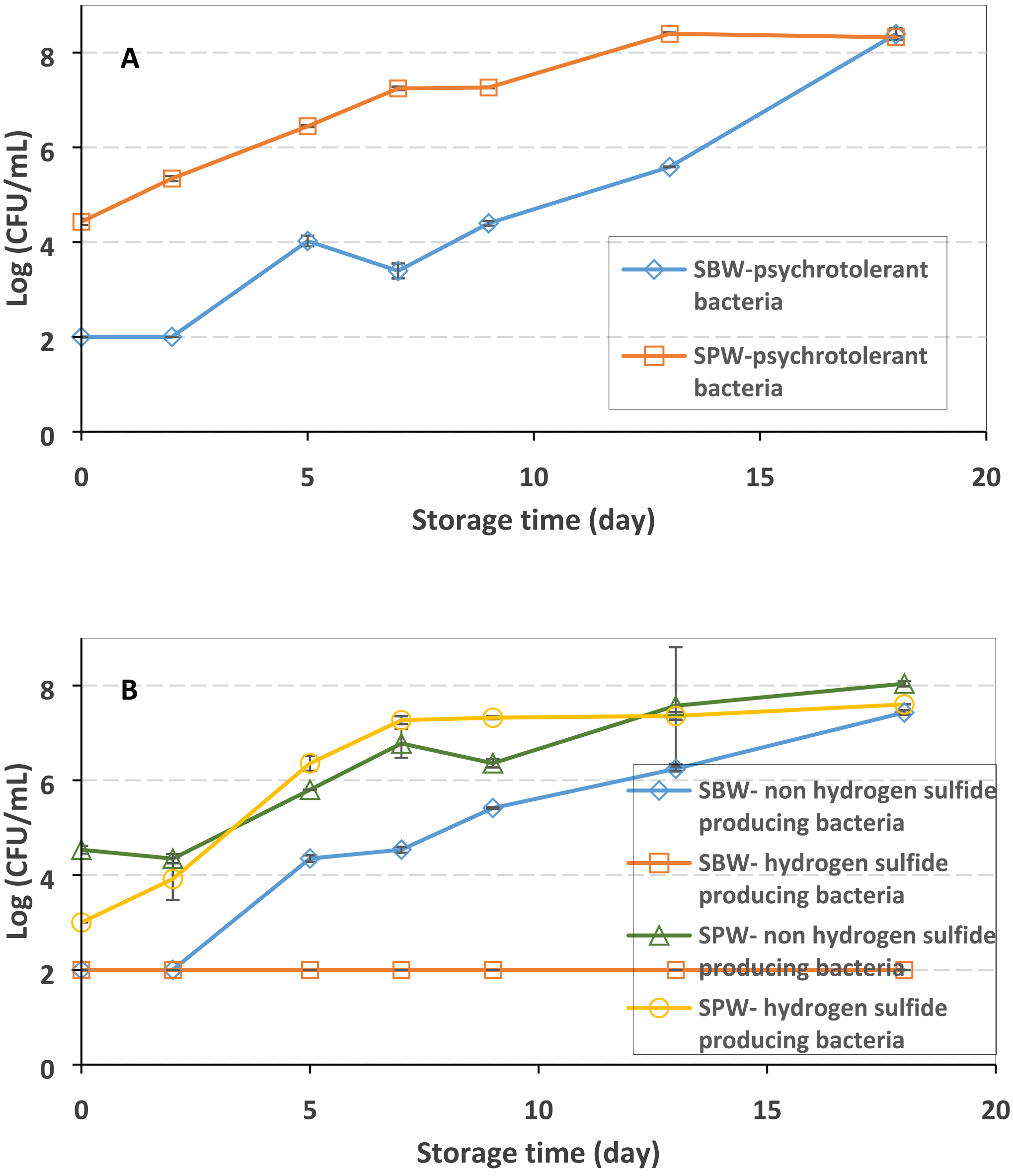

Figure 2S. Analyses of psychrotolerant bacteria (A), hydrogen sulfide and non-hydrogen sulfide producing bacteria (B) in SBW and SPW during cold storage (4°C). Data points show average ±SD, n=2.

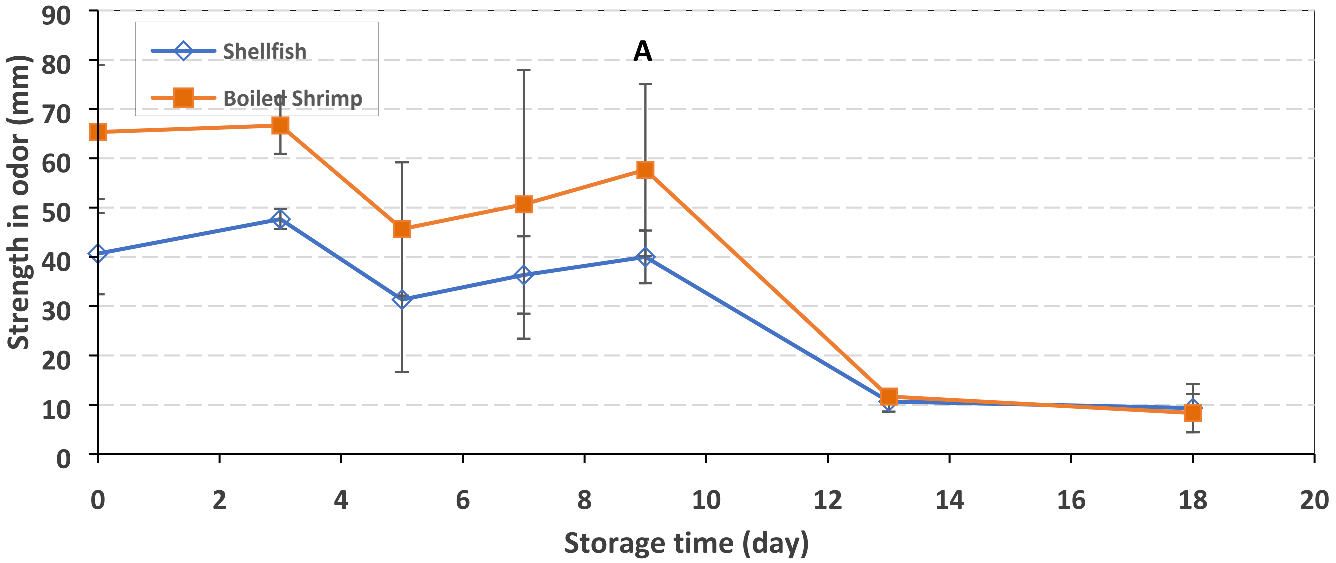

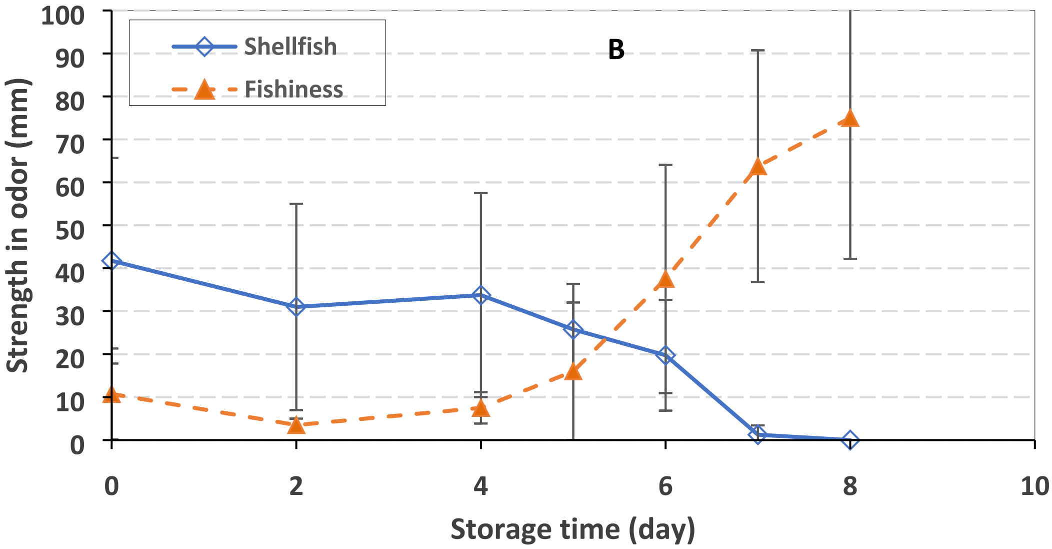

Figure 3S, Sensory characteristics of SBW (A) and SPW (B) during cold storage (4°C). Data points show average ±SD, n=5.
